# Editorial: Novel fMRI techniques and analysis methods for enhanced detection of functional disorders

**DOI:** 10.3389/fnins.2024.1466071

**Published:** 2024-08-07

**Authors:** Seong Dae Yun, Sung Suk Oh, Min Cheol Chang

**Affiliations:** ^1^Institute of Neuroscience and Medicine 4 (INM-4), Forschungszentrum Jülich, Jülich, Germany; ^2^Medical Device Development Center, Daegu-Gyeongbuk Medical Innovation Foundation (K-MEDI hub), Daegu, Republic of Korea; ^3^Department of Rehabilitation Medicine, College of Medicine, Yeungnam University, Daegu, Republic of Korea

**Keywords:** advanced analysis, clinical diagnosis, data pre-processing, functional MRI (fMRI), functional connectivity, high-resolution, imaging technique, paradigm design

The advent of functional magnetic resonance imaging (fMRI) has introduced new possibilities for the non-invasive investigation of neural activities in the *in vivo* brain. The general availability and unique advantages of fMRI over other neuroimaging modalities, such as unrivalled *in vivo* spatiotemporal resolution and the absence of radiation exposure, have led to its widespread use in enormous neuroimaging studies. Moreover, various fMRI acquisition techniques, paradigm designs, data processing strategies, and analysis methodologies have been developed to effectively detect and accurately characterise neural activities. These approaches have advanced our understanding of brain function and its underlying neurophysiological mechanisms, thereby facilitating their use in the clinical diagnosis of brain disorders. The aforementioned subject is the main focus of our Research Topic, which covers fMRI studies exploring new insights into the delineation of brain activity and clinical evaluation through recent developments in the field.

The remarkable advances in current fMRI techniques allow the acquisition of functional signals with a submillimeter voxel size. This innovation enables the recording and interpretation of neural signals at the laminar or columnar level, providing insights into the fundamental neural circuitry and the hierarchical processing between brain areas at different cortical depths. This topic was addressed in a study by Pais-Roldán et al., which investigated cortical depth-dependent activity under the resting-state condition using an ultra-high resolution fMRI technique. The employed technique (Yun et al., 2022) was shown to be capable of providing 0.63 mm isovoxels with near whole-brain coverage, allowing the examination of various networks throughout the brain in a single fMRI session. Specifically, the authors demonstrated differences in the organisation of depth-dependent functional connectivity across different brain networks from healthy subjects; for instance, the default mode network is primarily maintained by intermediate and superficial layers of the cortex, whereas the executive control network predominantly engages deeper cortical layers. These findings suggest that the investigation of the brain's overall functional connectivity at the cortical depth level, facilitated by a novel fMRI technique, can effectively be deployed in the clinical assessment of psychiatric disorders, as they potentially involve disturbances of large-scale networks (Ishida et al., 2023).

Notwithstanding the advances in fMRI techniques, the intrinsic or imaging sequence-specific source of artefacts, such as head motion, low-frequency drifts, and spatial distortion, may substantially hinder the accurate analysis of functional data. For effective elimination of these artefacts, various pre-processing methods have been presented with an appropriate selection of the steps. Yue et al. have proposed a novel iterative data-driven whitening pre-processing step, which outperforms conventional methods in terms of elimination of serial correlation and type-I error in fMRI time-series data, while preserving higher statistic power. This approach can be applied for both long and short TR regimes, addressing the limitations of the conventional low-order model, which is not effective for short TR cases. This contribution is particularly important for fMRI studies focusing on the investigation of dynamic functional connectivity, where a sub-second TR is typically used for enhanced and consistent detectability (Sahib et al., 2018).

Following the pre-processing of fMRI data, the application of an appropriate analysis method is crucial for accurately extracting hemodynamic signals. This can range from classic statistical tests focusing on signal changes at individual voxels to more complex metrics exploring spatial and temporal patterns of neural activity across disparate brain areas. Xu et al. addressed the limitation of static fMRI analysis in the clinical assessment of post-stroke aphasia (PSA) and suggested the investigation of dynamic alterations in spontaneous neural activity. The results demonstrated an increased dynamic amplitude of low-frequency fluctuations in the cerebellar network from the PSA group, a finding not previously observed in static studies. Moreover, while most previous studies have focused on subacute and chronic phases of PSA, the authors have contributed to understanding the dynamic properties during the acute phase, potentially offering novel insights into recovery mechanisms and the development of enhanced treatments. In another interesting article, Chen et al. reviewed 31 studies that utilised machine learning (ML) to classify major depressive disorder from resting-state fMRI data. Accurate diagnosis and prediction of psychiatric disorders have been challenging in clinical practise, despite the widespread use of resting-state fMRI. However, this article performed a meta-analysis of the data presented in those 31 studies and demonstrated the high diagnostic performance of ML in distinguishing the disorder from healthy controls. The use of ML has gained significant popularity in neuroimaging studies for diverse purposes, such as image reconstruction, reducing image artefacts, and classifying regions of interest. Specifically, in fMRI analysis, ML has been increasingly exploited in multivariate pattern analysis, which investigates neural activity patterns from multiple regions that are collectively associated with certain cognitive functions. This analysis offers increased sensitivity to spatially distributed effects compared to the conventional univariate method, making it suitable for studies on subclinical populations with mild alterations (Portugal et al., 2023).

In addition to the methodological developments described above, experimental design in fMRI also serves an important role in the derivation of desirable functional signals. This feature was highlighted in the work by Manuel et al., which successfully depicted individual glucose-sensitive hypothalamic pathways in a human subject for the first time using fMRI with glucose challenges. To avoid the potential inter-individual variability in group studies that most fMRI studies focus on, the authors instead examined the n-of-1 trial responses obtained from a single subject and demonstrated its effectiveness in comparison to group results.

In summary, the articles presented in our Research Topic have made outstanding contributions to advancing fMRI methodologies in various ways and have shown great potential for understanding functional physiology and enhancing clinical diagnosis. The utility of fMRI in clinical applications has been increasingly leveraged as a biomarker for early detection and tracing disease progression through longitudinal studies (Renga, 2022), which necessitate reliable and reproducible fMRI methods. As a future direction, the need of this community fosters continued development of robust fMRI techniques, offering superior spatiotemporal resolution beyond the current state-of-the-art, coupled with sophisticated experimental design, data processing, and analysis schemes. This development will further benefit from the integration of ML techniques.

## Author contributions

SY: Conceptualization, Supervision, Writing – original draft, Writing – review & editing. SO: Writing – review & editing. MC: Writing – review & editing.

## References

[B1] Ishida T. Nakamura Y. Tanaka S. C. Mitsuyama Y. Yokoyama S. Shinzato H.. (2023). Aberrant large-scale network interactions across psychiatric disorders revealed by large-sample multi-site resting-state functional magnetic resonance imaging datasets. Schizophr Bull. 49, 933–943. 10.1093/schbul/sbad022 36919870 PMC10318885

[B2] Portugal L. C. L. Ramos T. C. Fernandes O. Bastos A. F. Campos B. Mendlowicz M. V.. (2023). Machine learning applied to fMRI patterns of brain activation in response to mutilation pictures predicts PTSD symptoms. BMC Psychiatry 23:719. 10.1186/s12888-023-05220-x 37798693 PMC10552290

[B3] Renga V. (2022). Brain connectivity and network analysis in amyotrophic lateral sclerosis. Neurol Res Int. 2022:1838682. 10.1155/2022/1838682 35178253 PMC8844436

[B4] Sahib A. K. Erb M. Marquetand J. Martin P. Elshahabi A. Klamer S.. (2018). Evaluating the impact of fast-fMRI on dynamic functional connectivity in an event-based paradigm. PLoS ONE 13:e0190480. 10.1371/journal.pone.0190480 29357371 PMC5777653

[B5] Yun S. D. Pais-Roldan P. Palomero-Gallagher N. Shah N. J. (2022). Mapping of whole-cerebrum resting-state networks using ultra-high resolution acquisition protocols. Hum. Brain Mapp. 43, 3386–3403. 10.1002/hbm.25855 35384130 PMC9248311

